# Achondroplasia natural history study (CLARITY): 60-year experience in orthopedic surgery from four skeletal dysplasia centers

**DOI:** 10.1186/s13023-023-02738-x

**Published:** 2023-06-06

**Authors:** Nickolas J. Nahm, W. G. Stuart Mackenzie, William G. Mackenzie, Ethan Gough, S. Shahrukh Hashmi, Jacqueline T. Hecht, Janet M. Legare, Mary Ellen Little, Peggy Modaff, Richard M. Pauli, David F. Rodriguez-Buritica, Maria Elena Serna, Cory J. Smid, Julie Hoover-Fong, Michael B. Bober

**Affiliations:** 1 Nemours Children’s Hospital, DE, Wilmington, DE USA; 2 Bloomberg School of Public Health, Johns Hopkins University, Baltimore, MD USA; 3 Greenberg Center for Skeletal Dysplasias, Department of Genetic Medicine, Johns Hopkins University, Baltimore, MD USA; 4 Department of Pediatrics, McGovern Medical School UTHealth, Houston, TX USA; 5 Department of Pediatrics, University of Wisconsin School of Medicine and Public Health, Madison, WI USA; 6 Department of Orthopedics, Gillette Children’s Specialty Healthcare, Saint Paul, MN USA

**Keywords:** Achondroplasia, Spinal stenosis, Thoracolumbar kyphosis, Genu varum

## Abstract

**Background:**

The purpose of this study was to describe the frequency and risk factors for orthopedic surgery in patients with achondroplasia. CLARITY (The Achondroplasia Natural History Study) includes clinical data from achondroplasia patients receiving treatment at four skeletal dysplasia centers in the United States from 1957 to 2018. Data were entered and stored in a Research Electronic Data Capture (REDCap) database.

**Results:**

Information from one thousand three hundred and seventy-four patients with achondroplasia were included in this study. Four hundred and eight (29.7%) patients had at least one orthopedic surgery during their lifetime and 299 (21.8%) patients underwent multiple procedures. 12.7% (n = 175) of patients underwent spine surgery at a mean age at first surgery of 22.4 ± 15.3 years old. The median age was 16.7 years old (0.1–67.4). 21.2% (n = 291) of patients underwent lower extremity surgery at a mean age at first surgery of 9.9 ± 8.3 years old with a median age of 8.2 years (0.2–57.8). The most common spinal procedure was decompression (152 patients underwent 271 laminectomy procedures), while the most common lower extremity procedure was osteotomy (200 patients underwent 434 procedures). Fifty-eight (4.2%) patients had both a spine and lower extremity surgery. Specific risk factors increasing the likelihood of orthopedic surgery included: patients with hydrocephalus requiring shunt placement having higher odds of undergoing spine surgery (OR 1.97, 95% CI 1.14–3.26); patients having a cervicomedullary decompression also had higher odds of undergoing spine surgery (OR 1.85, 95% CI 1.30–2.63); and having lower extremity surgery increased the odds of spine surgery (OR 2.05, 95% CI 1.45–2.90).

**Conclusions:**

Orthopedic surgery was a common occurrence in achondroplasia with 29.7% of patients undergoing at least one orthopedic procedure. Spine surgery (12.7%) was less common and occurred at a later age than lower extremity surgery (21.2%). Cervicomedullary decompression and hydrocephalus with shunt placement were associated with an increased risk for spine surgery. The results from CLARITY, the largest natural history study of achondroplasia, should aid clinicians in counseling patients and families about orthopedic surgery.

**Supplementary Information:**

The online version contains supplementary material available at 10.1186/s13023-023-02738-x.

## Background

Achondroplasia, with an incidence of 1 in 20,000 to 30,000 births, is the most common form of skeletal dysplasia [1, 2]. The dysplasia is caused by a gain of function pathogenic variant in fibroblast growth factor receptor-3 (*FGFR3*) [3, 4]. Signaling through FGFR3 inhibits chondrocyte proliferation and differentiation leading to abnormal cartilaginous bone growth and a short-limbed dysplasia [5]. Achondroplasia results from mutations which are monoallelic, autosomal dominant, 100% penetrant and approximately 80% de novo [6, 7]. The gain of function *FGFR3* pathogenic variant leads to physeal pathology and abnormal endochondral ossification [8].

Skeletal manifestations of achondroplasia include spondylometaphyseal dysplasia, rhizomesomelia, thoracolumbar kyphosis (TLK), spinal stenosis, genu varum, and trident hands [9-13]. Common sequelae include foramen magnum stenosis, obstructive sleep apnea, obesity, and recurrent ear infections [5, 14, 15]. Radiographic examination of the spine reveals short pedicle length with decreased interpedicular distance in the lumbar spine [13]. Imaging of the lower extremities demonstrates V-shaped epiphyses with apex toward the metaphysis [13]. Histologic abnormalities are seen in the proliferative zone of the physis [8, 9].

Despite the knowledge that spinal and lower extremity deformities frequently require surgery, the timing and type of orthopedic intervention is not fully described. One of the largest studies previously addressing the frequency of surgical intervention in patients with achondroplasia included less than 200 patients [16]. The A**c**hondrop**l**asia N**a**tu**r**al H**i**s**t**ory Stud**y** (CLARITY) is a multicenter study of 1,374 patients with achondroplasia treated at four skeletal dysplasia centers across six decades [17]. One of the primary aims of CLARITY is to delineate the frequency, temporal trends and risk factors for orthopedic surgery intervention in achondroplasia.

The major conditions requiring orthopedic procedures are spinal stenosis, TLK and lower extremity deformity [16]. Spinal stenosis results from combination of shortened pedicle length leading to a diminished sagittal canal length as well as decreased interpedicular distance leading to a diminished coronal canal length [9, 16, 18]. This altered pedicular anatomy results in decreased space available for the spinal cord. Furthermore, progressive thickening of the ligamentum flavum and facet hypertrophy can further compromise the space available for the cord [19]. As a consequence, patients may develop symptoms of pain, neurogenic claudication, long-track signs, decreased mobility and bowel and/or bladder dysfunction [11, 19, 20]. Symptomatic spinal stenosis may ultimately require decompression and/or fusion [20].

TLK is present in all infants with achondroplasia and is thought to be a result of hypotonia and other factors [5, 21]. The majority of children improve spontaneously, with 15% showing improvement by walking age and 60% by 1 year after walking [22, 23]. Borkhuu et al. reported that progressive kyphosis was significantly associated with developmental motor delay (walking after 24 months of age compared to 18 months), apical vertebral wedging and apical vertebral translation [24]. Estimates of patients with achondroplasia who will develop symptomatic, fixed deformities requiring surgery range from 10–30% [5, 21].

The lower extremity deformity in achondroplasia is often referred to as bowing or genu varum, but it is in fact, a complex combination of multiple factors including lateral, dynamic instability of the knee; distal femur, proximal and distal tibial varus; internal tibial torsion; fibular overgrowth and tibial recurvatum [5, 12, 25]. At least one study by Ain et al., suggested that genu varum may be more common in males than females [26]. When these factors combine to result in leg pain and compromised physical function, realignment procedures are indicated [12]. Historically, the lower extremity deformities were addressed with osteotomies, but more recently less invasive guided growth procedures are emerging including tension band plates and screw hemi-epiphysiodesis [28]. These newer procedures may lead to a change in the timing and indications for surgery. Previous studies suggested that approximately 1 in 4 patients with achondroplasia will require surgical intervention for genu varum [5, 16, 27].

Despite the known requirement for orthopedic procedures, the frequency and timing of orthopedic intervention in patients with achondroplasia are not well established. This study provides important new information and offers unique insight into the course of treatment across the lifespan in a large cohort of patients with achondroplasia.

## Results

### Surgical overview

One thousand three hundred and seventy-four subjects with achondroplasia, constituting the Primary Achondroplasia Cohort (PAC), comprise the CLARITY population, with a mean age at the last encounter of 15.4 ± 13.9 years and a median age at the last encounter of 11.9 years (5.9–19.7) (Table 1) [17]. Overall, 408 patients (29.7%) had at least one orthopedic surgery during their lifetime. At the time of the first procedure, the mean age was 13.7 ± 12.7 years with a median age of 9.9 years (0.1–62.7). One hundred seventy-five individuals underwent one or more spine procedures alone. Their mean age was 22.4 ± 15.3 years with a median age of 16.7 years (0.1–62.7). 291 individuals underwent one or more lower extremity procedures alone at a mean age of 9.9 ± 8.3 years and a median age of 8.2 years (0.2–57.8). Fifty-eight individuals had both a spinal and lower extremity procedure. In this group, the mean age for the second procedure was 13.8 ± 11.4 years with a median age of 12.8 years (1.1–57.8). The most common spinal procedure was decompression, while the most common lower extremity procedure was osteotomy.

**Table 1:** Types of orthopedic surgeries performed

	Number of procedures	Number of subjects *		Age, years**	
		With at least one procedure	With multiple procedures	Mean ± SD	Median (minimum–maximum)
Any orthopaedic procedure	1109	408	299	13.7±12.7	9.9 (0.1–62.7)
Both spine and lower extremity procedure	267	58	58	13.8 ± 11.4	12.8 (1.1–57.8)
Spine procedures	425	175	116	22.4±15.3	16.7 (0.1–67.4)
Any laminectomy	235	126	54	25.5±115.6	20.6 (0.1–67.6)
Cervical laminectomy (below C2)	38	29	6	24.6 ± 18.3	21.0 (0.1–64.4)
Thoracic laminectomy	92	67	16	27.3 ± 16.5	21.7 (3.4–65.8)
Lumbosacral laminectomy	141	109	22	26.7 ± 15.2	23.8 (2.0–67.4)
Spinal fusion	142	106	24	21.0 ± 15.1	15.6 (0.8–65.8)
Other	12	11	1	13.0 ± 11.2	11.5 (3.1–42.4)
Lower extremity procedures	684	291	199	9.9±8.3	8.2(0.2–57.8)
Osteotomy	434	200	163	9.1 ± 5.8	7.9 (1.1–36.9)
Tension band plating for guided growth	27	23	4	8.6 ± 2.9	8.9 (4.0–12.3)
Fibulectomy	26	22	4	6.3 ± 2.4	6.0 (2.5–11.4)
Limb lengthening	24	17	5	9.9 ± 5.0	9.6 (0.2–20.5)
Epiphysiodesis	13	11	1	7.8 ± 2.9	7.6 (4.0–12.4)
Hip replacement	4	3	1	46.1 ± 7.6	42.4 (41.1–54.8)
Implant removal	87	73	11	9.9 ± 5.1	10.7 (2.4–27.8)
Other	69	58	9	15.3±12.4	13.6(1.0–57.8)

^*^The procedures are not mutually exclusive, SD, standard deviation

^**^Ages are for first time procedures

The types of spine and lower extremity procedures performed are displayed with the number of patients undergoing at least one of these procedures and the number of patients undergoing multiple procedures. Decompression was the most common spine surgery, and osteotomy was the most common lower extremity surgery. The age at which the first surgery was performed is also demonstrated. Spine procedures were performed later in life compared to lower extremity procedures

**Fig. 1:**
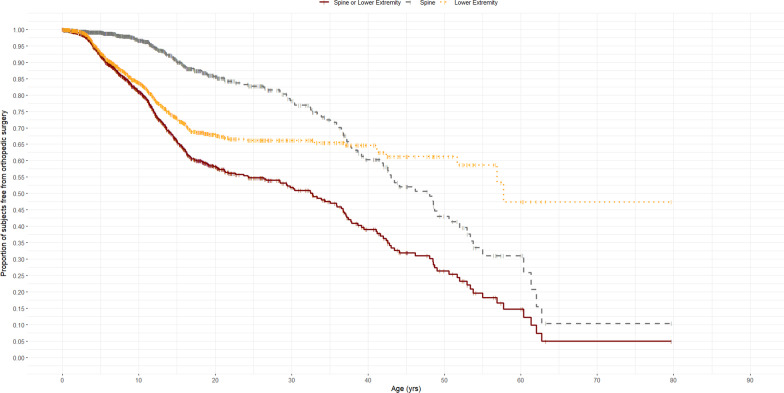
Kaplan Meier curve of patients undergoing any orthopaedic, lower extremity or spine surgery by age. These data represent cumulative probability of a surgery (y-axis) as a function of age (x-axis). At 10 years of age, 19.0% of patients had an orthopaedic procedure with 16.2% having a lower extremity surgery and 3.4% with spine surgery. At 20 years of age, 41.8% of patients had an orthopaedic procedure with 32.0% having a lower extremity surgery and 14.4% with spine surgery. At 40 years of age, 60.9% had an orthopaedic procedure with 35.3% having a lower extremity surgery and 39.6% with spine surgery. At 60 years of age, 85.3% had an orthopaedic procedure with 52.6% having lower extremity surgery and 68.9% with spine surgery. At 80 years of age, 95.1% had an orthopaedic procedure with 52.6% having lower extremity surgery and 89.6% with spine surgery

To account for the age of the subject at the last observation, Kaplan Meier analysis of the 1365 patients with complete surgical history is shown in Fig. 1. At 10 years of age, 120 of 635 (19.0%) had either type of orthopedic procedure, with 103 of 635 (16.2%) being lower extremity procedures. At 20 years of age, this increased to 41.8% (87 of 212) patients with the majority of these procedures (68 of 212; 32%) involving the lower extremities. After the age of 20, further increases were predominately spinal procedures, and by 60 years of age, 85.3% of 8 patients who were known to reach that age had an orthopedic procedure with a shift to spine procedures being more common.

### Spine surgery overview

**Fig. 2:**
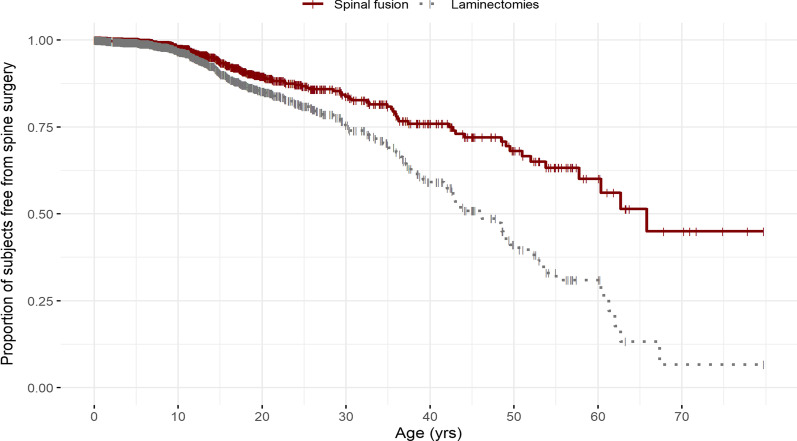
Kaplan Meier curve of patients undergoing laminectomies and spinal fusions by age. These data represent cumulative probability of a surgery (y-axis) as a function of age (x-axis). At 10 years of age, 3.4% of patients had a laminectomy and 2.4% had a fusion surgery. At 20 years of age, 14.8% of patients had a laminectomy and 10.5% had a fusion surgery. At 40 years of age, 40.9% had a laminectomy and 24.1% had fusion surgery. At 60 years of age, 69.1% had a laminectomy and 39.9% had a fusion. At 80 years of age, 93.4% had a laminectomy with 55.0% had fusion surgery

One hundred and seventy-five patients (12.7%) underwent 425 spine procedures (Table 1). Spine procedures were classified as fusion and/or laminectomy of the cervical (below C2), thoracic or lumbosacral levels. Laminectomy of C1 and C2 are excluded here but have been published [29]. The most common spine surgery was decompression with 152 patients undergoing 271 laminectomy procedures in the cervical, thoracic, and lumbosacral spine. Lumbar laminectomies were most frequent with L3 being the most common level. Thoracic and cervical regions followed in frequency, with T11 and T12 being the most common thoracic levels. The mean age for all laminectomies was 25.5 ± 15.5 years with a median age of 20.6 (0.1–67.6). One hundred and six patients underwent 142 fusion procedures with a mean age of 21.0 ± 15.1 years and a median age of 15.6 years (0.8–65.8). Laminectomy was performed in patients with spinal stenosis, and fusion was performed in those patients who had a risk of progressive kyphosis after decompression, as determined by the surgeon. Most surgeries classified as ‘other’ spine procedures are implant/hardware revision or removal. Spine surgery continued throughout the lifespan (Fig. 2).

### Lower extremity surgery overview

Two hundred and ninety-one patients (21.2%) underwent 684 lower extremity procedures (Table 1). Lower extremity surgical procedures included osteotomy, guided growth, epiphysiodesis, fibular shortening, limb lengthening, and total hip arthroplasty. The most common lower extremity surgery was osteotomy with 200 patients undergoing 434 lower extremity osteotomies. The mean age for osteotomy was 9.1 ± 5.8 years with a median age of 7.9 years (1.1–36.9). Twenty-three patients underwent 27 guided growth tension band plating. The mean age for guided growth tension band plating was 8.6 ± 2.9 years with a median age of 8.9 years (4.0–12.3). Of the 27 procedures, none were performed in the ‘before 1980s’ birth cohort. One was performed in the 1980s birth cohort with the numbers increasing to 6, 16 and 4 respectively in the 1990s, 2000s and 2010s birth cohorts. The other category of lower extremity procedures included arthroscopic discoid meniscus saucerization, fixator removal, and arthrograms. All patients underwent lower extremity surgery before 60 years of age (Fig. 1).

### Risk factors for surgery

Risk factors associated with having any spine, lower extremity or both types of surgery are shown in Table 2. There was no difference in the odds of these surgeries given the sex of the subject. There was a 1.85 times greater odds of spine surgery in this cohort if a cervicomedullary decompression (CMD) had been performed previously (p =  < 0.01) but there was no increased risk for any lower extremity surgery (p = 0.8). Hydrocephalus requiring shunting increased the risk for both a spine surgery (OR 1.97; p = 0.011), and for both a spine and lower extremity procedure (OR 1.94; p =  < 0.01), but not a lower extremity procedure alone (OR1.46; p = 0.118). Of all the risk factors evaluated, a history of both shunting and CMD was associated with the highest risk for subsequent additional spinal surgery (OR 2.268; p = 0.017). The risk for a lower extremity procedure (1.93, p = 0.031) or both types of surgery (2.38, p < 0.01) were also increased. The use of CPAP to treat obstructive sleep apnea (OSA) increased the odds of any orthopedic surgery combined (OR 1.5, p = 0.02), but not individually for spine procedures or lower extremity procedures. If an individual underwent a surgery in the spine or lower extremity, the odds of them needing a subsequent procedure in the other domain was increased 2.05 times (p =  < 0.01).

**Table 2:** Risk factors for the first orthopaedic surgery

	Spine surgery		Lower extremity surgery		Both spine and lower extremity surgery	
	OR (95% CI)	p	OR (95% CI)	p	OR (95% CI)	p
Gender (male:female)	1.12 (0.82–1.54)	0.48	1.11 (0.85–1.44)	0.44	1.12 (0.89–1.41)	0.35
CMD	1.85 (1.30–2.63)	< 0.01	0.96 (0.69–1.32)	0.80	1.10 (0.83–1.46)	0.50
Hydrocephalus requiring shunt	1.97 (1.14–3.26)	= 0.01	1.46 (0.89–2.31)	0.118	1.95 (1.28–2.97)	< 0.01
CMD and hydrocephalus requiring shunt (on different dates)	2.26(1.11–4.28)	= 0.017	1.93 (1.04–3.46)	0.031	2.38 (1.35–4.18)	< 0.01
OSA on CPAP	1.42 (0.90–2.24)	0.13	1.32 (0.90–1.93)	0.16	1.50 (1.06–2.12)	0.02
Lower extremity surgery	2.05 (1.45–2.90)	< 0.01	–	–	–	–
Spine surgery	–	–	2.05 (1.45–2.90)	< 0.01	–	–
Height	1.03 (1.02–1.03)	< 0.001	0.99 (0.99–1.00)	0.149	1.01 (1.00–1.01)	0.027
Weight	1.04 (1.02–1.05)	< 0.001	0.98 (0.96–1.00)	0.023	1.01 (1.00–1.02)	0.034
OFC	1.06 (1.02–1.09)	< 0.001	0.98 (0.96–1.00)	0.050	1.00 (0.98–1.02)	0.826

The relationship between patient height, weight, and occipital frontal circumference (OFC) and the need for orthopedic surgery was also evaluated (Table 2). Modest statistically significant increases in the odds ratios (OR 1.03, p =  < 0.001 and OR 1.01, p = 0.027) were found for increasing height related to spine or spine and lower extremity surgery, respectively (the taller the more likely). No associated risk was seen between height and lower extremity surgery. Weight also showed a modest significant increase in the odds ratios for spine or spine and lower extremity surgery, respectively (OR 1.04, p =  < 0.001 and OR 1.01, p = 0.034). Increasing weight however, slightly decreased the likelihood of requiring a lower extremity surgery (OR 0.98, p = 0.023). In this group of risk factors, OFC increased the risk for spine surgery (OR 1.06, p =  < 0.001) but not for lower extremity procedures or both lower extremity and spine surgery.

### Additional spine surgery results

#### Indications

**Fig. 3:**
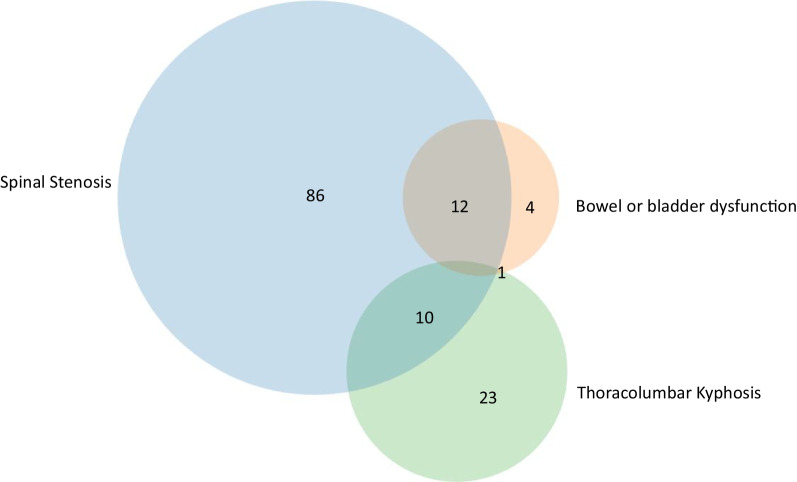
Indications for first time spine surgery. A Venn diagram is used to show the most common indications and their overlap for spine surgery. The most common indications were symptomatic spinal stenosis followed by thoracolumbar kyphosis. Additional, less frequent indications are not represented (n = 37)

The most common indications for first time spine surgery included symptomatic spinal stenosis (n = 108), TLK (n = 34), and bowel or bladder dysfunction (n = 17, Fig. 3). Symptomatic spinal stenosis and bowel and bladder dysfunction were both present in 12 patients while symptomatic spinal stenosis and TLK were both present in 10 patients. One patient had bowel or bladder dysfunction and TLK. When the indication for a spinal decompression surgery was TLK compared to all other potential indications, the median ages were significantly different at surgery was 13.2 years and 22.7 years (p = 0.026), respectively.

#### Outcomes

118 initial spine surgeries were reported to have had good outcomes, and 13 had no improvement (Table 3). The most common complication after spine surgery was paresthesia, occurring after 7 procedures followed by infection in 3 patients and pseudarthrosis in 1 patient. Four surgical events were associated with repeat surgery within one year of surgery. Four surgical events were associated with hospitalization within one month after surgery.

**Table 3:** Outcomes and complications for first time spine surgery and repeat spine surgery

Outcomes	First spine surgery	Repeat spine surgery
Good outcome	118	161
No improvement	13	11
Re-hospitalized within 1 month of surgery	4	2
Repeated procedure within 1 year of surgery	4	6
*Complications*		
Anesthetic complication	2	0
Bleeding	1	0
Poor wound healing	3	1
Pseudarthroses	1	4
Paresthesia	7	7
Dislocation	0	0
Pulmonary complication	1	1
Infection	3	6
Worsened medical status	2	2
Other	21	25
Unknown	17	23

#### By decade

Patients were stratified by birth decade and incidence ratios were calculated for each birth decade for laminectomies (Table 4). The highest number of surgeries per 1000 patient years of follow-up was seen among the oldest patients, born before 1980. When an incidence ratio was calculated with the 2010 birth decade cohort used as the reference, those born before 1980 were 7.57 times more likely to have had a spinal procedure. The 1980s and 1990s birth decade cohorts were similar at approximately 5 times more likely.

**Table 4:** Incidence of laminectomy procedures by birth cohort

Birth Cohort	Total patients	Patients with laminectomies surgery N (%)	Age at first laminectomy surgery Mean ± SD	Total number of laminectomy surgeries	Years contributed	Surgeries per 1000 person years	Incidence rate ratio (95% CI)
2010–date	239	2 (0.8)	2.99 ± 1.90	2	811.62	2.46	Reference
2000–2009	356	7 (2.0)	10.18 ± 3.80	10	3481.37	2.87	1.17 (0.65 – 2.11)
1990–1999	314	38 (12.1)	20.97 ± 4.19	57	4639.11	12.29	5.00 (3.01–8.30)
1980–1989	231	32 (13.9)	27.60 ± 6.08	50	4085.37	12.24	4.98 (2.99–8.29)
< 1980	234	73 (31.2)	50.61 ± 14.56	152	8158.95	18.63	7.57 (4.61–12.44)
TOTAL	1374	152 (11.1)		271	21,176.42		

Incidence was represented by person-year (surgeries per 1000 years) and the incidence rate ratio, which standardized the number of patients and years contributed in each cohort. A total of 271 laminectomy procedures were performed in 152 patients. Patients born before 1980 had the highest incidence rate ratio for laminectomy surgeries

#### By center

Kaplan Meier analysis of the probability of undergoing laminectomies or fusion surgeries independently of each other is shown in Fig. 2. Starting at approximately 15 years of age, there is a divergence in the frequency of these procedures with laminectomies occurring more frequently in older ages. Kaplan Meier curves for all spine procedures were generated by center (Additional file 1: Figure S1). Spine procedures performed at facilities that treat adults were performed later in life compared to duPont, which does not treat patients older than 35 years of age.

#### Repeat surgery

**Fig. 4:**
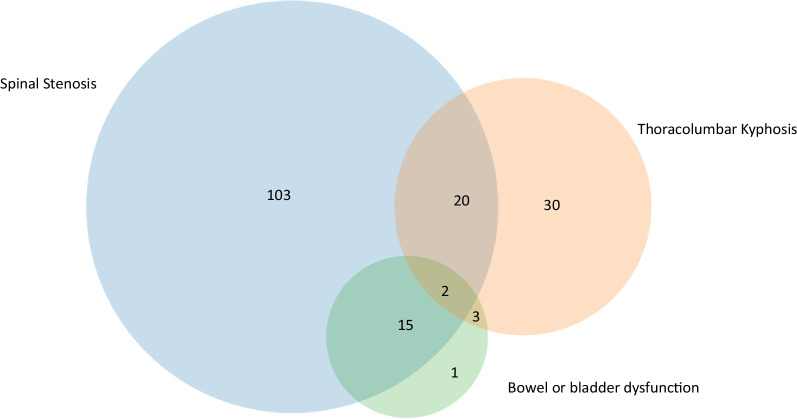
Indications for repeat spine surgery. A Venn diagram is used to show the most common indications and their overlap for repeat spine surgery. The most common indications were spinal stenosis followed by thoracolumbar kyphosis. Additional, less frequent indications are not represented (n = 72)

One hundred-sixteen subjects had multiple spinal procedures. The most common indications for repeat time spine surgery included spinal stenosis (n = 140), TLK (n = 55), and bowel or bladder dysfunction (n = 21) (Fig. 4). Both symptomatic spinal stenosis and bowel and bladder dysfunction were present in 15 patients while both symptomatic spinal stenosis and TLK were present in 20 patients. Two patients had all three indications and 3 had bowel or bladder dysfunction and TLK. Risk factors for repeat spine surgeries are shown in Table 5. The most significant risk for a repeat spine surgery was a prior cervicomedullary decompression (OR 2.54, p = 0.016) (Table 5). A modest, but statistically increased risk (OR 1.02, p = 0.001) was found for height and repeat spine surgery. Weight and OFC were also associated with significant increased risk for repeat spine surgery, respectively (OR 1.05, p < 0.007 and OR 1.05, p = 0.019).

**Table 5:** Risk factors for repeat orthopaedic surgery

	Repeat spine surgery		Repeat lower extremity surgery	
	OR (95% CI)	p	OR (95% CI)	p
Age at first surgery	1.01 (0.99–1.03)	0.507	0.98 (0.95–1.01)	0.142
Gender (male:female)	1.03 (0.55–1.94)	0.927	1.13 (0.69–1.87)	0.614
CMD	2.54 (1.23–5.64)	0.016	0.64 (0.35–1.17)	0.143
Hydrocephalus requiring shunt	1.66 (0.65–4.80)	0.314	0.74 (0.35–1.64)	0.450
CMD and hydrocephalus requiring shunt	2.40 (0.74–10.8)	0.185	0.99 (0.40–2.67)	0.983
OSA on CPAP	1.42 (0.90–2.24)	0.13	1.32 (0.90–1.93)	0.16
Prior lower extremity surgery	1.39 (0.71–2.78)	0.346		
Prior spine surgery			0.53 (0.30–0.97)	0.037
Height	1.02 (1.01–1.04)	0.001	0.99 (0.98–1.00)	0.094
Weight	1.05 (1.02–1.10)	0.007	0.99 (0.97–1.01)	0.435
OFC	1.07 (1.01–1.13)	0.019	1.00 (0.97–1.03)	0.838

One hundred sixty-one repeat spine surgeries were reported to be good outcomes, while 11 showed no improvement and 23 spine had unknown outcomes (Table 3). The most common complication after spine surgery was paresthesia (N = 7). Other complications included 6 infections and 4 pseudarthroses. Six surgical events after repeat surgery occurred within one year of surgery. Two surgical events required hospitalization within one month after surgery.

### Additional lower extremity surgery results

#### Indications

**Fig. 5:**
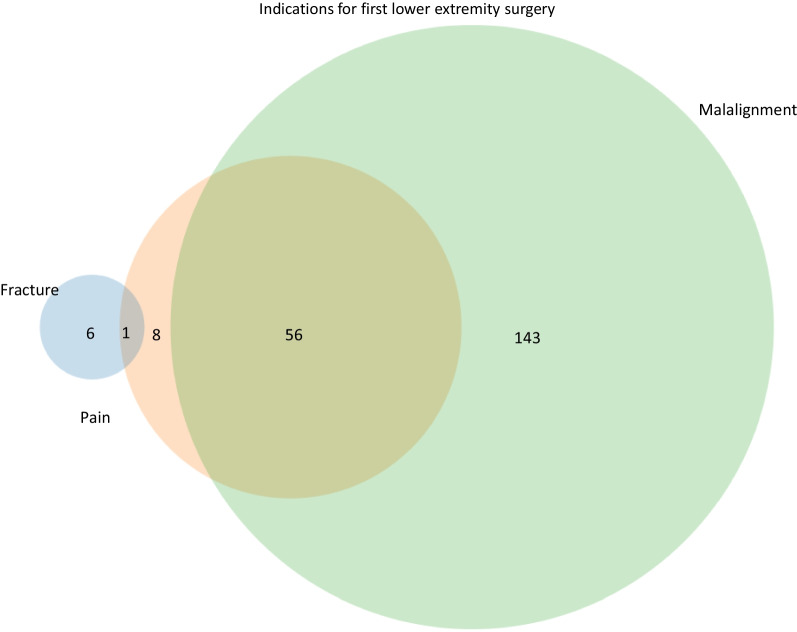
Indications for first time lower extremity surgery. A Venn diagram is used to show the most common indications and their overlap for lower extremity surgery. The most common indications were malalignment followed by pain. Additional, less frequent indications are not represented (n = 37)

The most common indications for the first lower extremity surgery were malalignment (n = 199) followed by pain (n = 65) and fracture (n = 7) (Fig. 5). Pain and malalignment together were indications for lower extremity surgery in 56 patients. One patient had pain and fracture.

#### Outcomes

Two hundred and one initial lower extremity surgeries were reported to have had good outcomes, (Table 6). The most common complication after lower extremity surgery was infection. None of initial surgical events were associated with repeat surgery within one year of surgery or a hospitalization one month after surgery (Table 6).

**Table 6:** Outcomes and complications after the first and repeat lower extremity procedures

Outcome	First lower extremity procedure	Repeat lower extremity procedure
Good outcome	201	284
No improvement	8	9
Rehospitalization within 1 month of surgery	0	0
Repeat procedure within 1 year of surgery	0	7
*Complications*		
Anesthetic complication	0	0
Bleeding	0	0
Poor wound healing	0	0
Nonunion	0	0
Paresthesia	0	1
Dislocation	0	0
Pulmonary complication	0	0
Infection	5	2
Worsened medical status	0	0
Other	22	23
Unknown	47	37

#### By decade

Patients were stratified by birth decade, and incidence ratios were calculated for each birth decade for lower extremity procedures (Table 7). The highest number of surgeries per 1000 patient years of follow-up was seen among patients born in the 1990s. This cohort had the highest incidence rate ratio for lower extremity procedures (3.35, 95% CI 3.15–5.47). When an incidence ratio was calculated with the 2010 birth decade cohort used as reference, those born before 1980 were 0.68 times less likely to have had a lower extremity procedure.

**Table 7:** Incidence of lower extremity procedures by birth cohort

Birth cohort	Total patients	Patients with lower extremity surgery N (%)	Age at first lower extremity surgery mean ± SD	Total number of lower extremity surgeries	Years contributed	Surgeries per 1000 patient years	Incidence rate ratio (95% CI)
2010–date	239	11 (4.6)	4.98 ± 1.03	17	813.96	20.89	Reference
2000–2009	356	65 (18.3)	7.17 ± 3.28	169	3140.49	53.81	2.58 (2.42–4.24)
1990–1999	314	100 (31.8)	8.91 ± 5.24	257	3674.43	69.94	3.35 (3.15–5.47)
1980–1989	231	68 (29.4)	9.63 ± 5.25	139	3189.94	43.57	2.09 (1.96–3.45)
< 1980	234	47 (20.1)	16.73 ± 13.79	103	7248.89	14.21	0.68 (0.64–1.14)
Total	1374	291 (21.2)		685	18,067.71		

Incidence was represented by person-year (surgeries per 1000 years) and the incidence rate ratio, which standardized the number of patients and years contributed in each cohort. A total of 685 lower extremity surgeries were performed in 291 patients. Patients born between 1990 and 1999 had the highest incidence rate ratio for lower extremity surgeries

#### By center

Kaplan Meier curves for lower extremity procedures were generated by center (Additional file 2: Figure S2). Most lower extremity surgeries were performed before 20 years of age at each center. In three of the centers, lower extremity procedures were also performed later in life between 40 and 60 years of age in nine patients. This is not true at duPont, which does not treat patients older than 35 years of age. In contrast, a second peak of lower extremity osteotomies was not observed in the older age groups. Several of the lower extremity surgeries which occurred at older ages included four total hip arthroplasties in three of the patients and bilateral total knee arthroplasties in one patient.

#### Repeat surgery

**Fig. 6:**
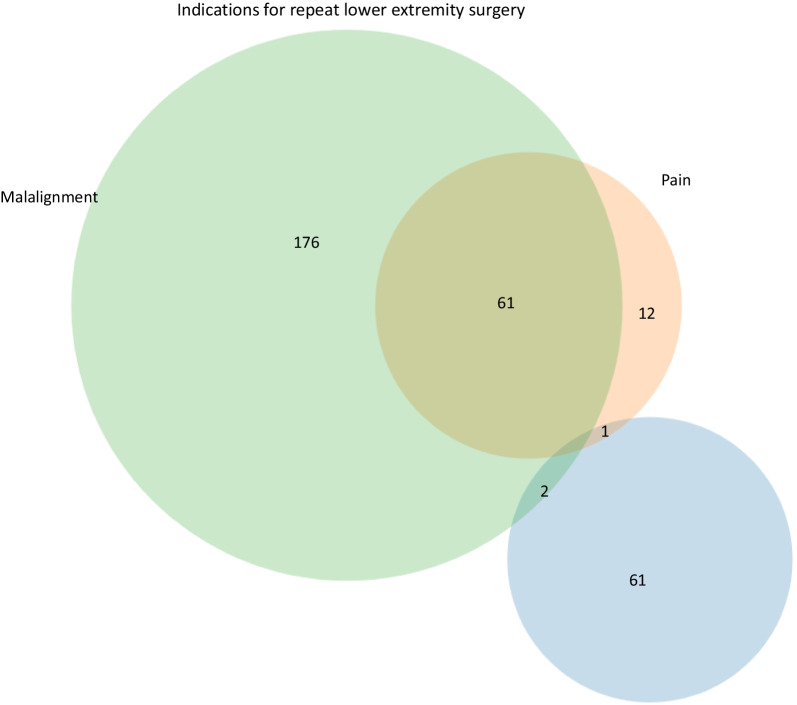
Indications for repeat lower extremity surgery. A Venn diagram is used to show the most common indications and their overlap for repeat lower extremity surgery. The most common indications were malalignment followed by pain. Additional, less frequent indications are not represented (n = 81)

One hundred-ninety-nine subjects had multiple lower extremity procedures. Malalignment was also the most common indication for repeat lower extremity surgery (n = 239) followed by pain (n = 74) and hardware removal (n = 64) (Fig. 6). Both malalignment and pain were present in 61 patients while malalignment and hardware removal were present in 2 patients. One individual had pain and required hardware removal. Risk factors for repeat lower extremity surgeries are listed in Table 5. Interestingly, having a prior spine surgery appears to be protective against having a repeat lower extremity procedure (OR 0.53, p = 0.037). No other significant risk factors were identified. No relationships were noted between HT, WT or OFC and the need for a repeat lower extremity surgery (Table 5).

Two hundred eight-four repeat lower extremity surgeries had good outcomes, and 9 surgeries reported no improvement (Table 6). The most common complication after a repeat lower extremity surgery was infection (n = 2). Seven surgical events required repeat surgery within one year of surgery. No surgical events required hospitalization within one month after surgery.

## Discussion

### Summary

CLARITY is the largest study reporting the results of a multicenter historical cohort of achondroplasia. This manuscript focuses on the orthopedic aspects of achondroplasia within the PAC. Orthopedic surgery was a common event in the care of achondroplasia with 408 patients (29.7%) undergoing at least one orthopedic intervention during their lifetime at a mean age of 13.7 years. This information is valuable for practicing orthopedic surgeons, as the new American Academy of Pediatrics care guidelines recommend orthopedic referrals for all children with achondroplasia [30]. Our data present the most detailed natural history to date, and should be used to facilitate improved care.

In the next largest study to report orthopedic procedures in patient with achondroplasia, Hunter et al. presented data on 193 patients with achondroplasia from one US center and five other centers across the world [16]. They reported that 21.6% of patients 20 years of age and older had undergone tibial osteotomy, similar to the frequency reported in our study. However, the frequency of spine surgery for stenosis in the prior study was 24.1% in patients 40 years of age and older. This rate of spine surgery was nearly twice the overall rate reported in our study (12.7%). In part, this difference may be due to a difference in the age distribution between the studies since our study population was younger, with only 7% of patients older than 40 years of age at the time of their last clinical contact.

However, in our subjects who reached 40 years of age, 39.6% had undergone a spinal surgery, which is 1.6 fold higher than the Hunter study.

### Risk factors

For the purposes of this analysis, we only included in this category, subjects who underwent CMD and shunt placement of different dates. This is due to the fact that during the 1980s and 1990s these procedures were often performed simultaneously. The subjects who underwent both procedures on the same date were included in the CMD group, as that was the primary surgical indication. The single largest risk factor associated with spinal, a lower extremity or or spinal and lower extremity surgery was previously having both a CMD for foramen magnum stenosis and a shunt placed for the treatment of hydrocephalus. The odds ratios were 2.26 (p =  < 0.017)1.93 (p-0.031) and 2.38 (p =  < 0.01), respectively.

In patients that had undergone a shunt placement for the treatment of hydrocephalus, the odds of requiring a spinal surgery, or both a spine and lower extremity procedure was increased, but not as great as when a shunt and CMD were performed. In patients that had undergone CMD alone, only the risk of spine surgery was increased (1.85, p =  < 0.01). The mechanism for narrowing of the foramen magnum and jugular foramina in *FGFR3-*related disorders is likely similar to that in the rest of the spine and may explain the increased odds of spinal decompression surgery in those with a prior shunt in the PAC [11, 19, 31]. These findings support the previous observations of the association between spinal stenosis and CMD in a much smaller achondroplasia population [11]. In that study of 44 patients undergoing surgery for spinal stenosis, Sciubba et al. found that more than 61% (27/44) also had CMD. Of these patients, 93% (25/27) had the CMD first and then additional spinal decompression later in life [11]. Although the proportion of patients in CLARITY with laminectomy who also underwent CMD was less (30.9%, 47/152), most patients had the CMD prior to their laminectomy (87.2%, 41/47).

OSA requiring CPAP was a risk factor for both types of orthopedic surgery (OR 1.50, 95% CI 1.06–2.12) but not spine or lower extremity surgery individually. OSA, in part, is the manifestation of abnormal cartilaginous development of the midface structures, but also associated with other factors such as increased weight. These pathologic processes also occur in the axial and appendicular spine and may interact with weight to accountfor this association.Future studies will be necessary to clarify this overall association and the temporaliy (i.e. did the requirement precede or follow the surgical procedures)..

### Spine

Compared to the general population, patients with achondroplasia undergo spine surgery much more frequently. In an analysis of the Nationwide Inpatient Sample of average stature individuals, the rate of lumbar fusion, thoracic fusion, and cervical fusion was estimated at 69.1, 7.9 and 51.9 per 100,000 patients [32]. In contrast, 7,700 per 100,000 patients (7.7%) with achondroplasia in our cohort underwent spine fusion which is over 100 times more common than the general population. This finding is likely related to the altered pedicular anatomy in achondroplasia, which predisposes to symptomatic spinal stenosis [18]. In average statured patients, stenosis remains a common indication for surgery but is more likely caused by other factors, including spondylosis [33]. Although spinal stenosis is treated with decompression, fusion is frequently necessary in patients with achondroplasia to prevent or address additional sagittal spine deformity [34].

The second most common indication for spine surgery was TLK (n = 34). TLK is present in most infants and young children with achondroplasia and usually resolves spontaneously with standing and walking (20). In those individuals who needed spine surgery for persistent TLK, the spine surgery occurred at a younger age (13.2 years) as compared to those who had all other indications (22.7 years). Unfortunately, CLARITY was unable to provide data regarding progression of TLK or the impact of bracing.

### Levels of procedures

Laminectomies were most commonly performed in the lumbar spine, most frequently at the L3 level. These findings are consistent with previous study of spinal stenosis in achondroplasia, which reported that the L2-3 level was most commonly decompressed [35]. In a study of pediatric patients, Scubbia et al. found that the most common level for decompression was at the thoracolumbar region (65%) followed by lumbar (20%) spine [11]. In our study, a significant number of laminectomies were performed in the cervical and thoracic spine, highlighting the importance of looking for stenosis beyond the lumbar spine and consideration of more than one focal area of stenosis in the same patient.

### Lower extremity

Genu varum is common among patients with achondroplasia and is related to a complex combination of multiple factors including lateral, dynamic instability of the knee; distal femur, proximal and distal tibial varus; internal tibial torsion; fibular overgrowth and tibial recurvatum [5, 12, 25]. It is frequently corrected with surgery [16]. Among patients with achondroplasia, lower extremity deformity involves the coronal, sagittal and transverse planes [12]. Historically, patients with genu varum were treated with corrective lower extremity osteotomies [36]. Although osteotomies are generally effective for addressing genu varum, these procedures are invasive. More recently, less invasive guided growth techniques utilizing tension band plates emerged to correct lower extremity deformity [37]. In a retrospective review of tension band plates among patients with skeletal dysplasia, Yilmaz et al. demonstrated correction in 34 of 38 valgus knees and 7 of 12 varus knees and concluded that this procedure was relatively safe and effective, even in young patients [38]. In another case series, McClure et al. reported the use of guided growth techniques in four patients with achondroplasia [28]. They found improvement in alignment in all patients; however, one patient did require subsequent osteotomies. They concluded that guided growth should be initiated at a younger age compared to patients without achondroplasia. Because guided growth was utilized in patients with skeletal dysplasia more recently, our data included only 23 patients undergoing 27 guided growth procedures. In our follow up of patients undergoing tension band plating for guided growth, none had a subsequent osteotomy. We anticipate that guided growth will be used with greater frequency, and future analysis should determine the impact of guided growth on the timing and frequency of lower extremity osteotomy as well as long-term physical function. Nevertheless, internal tibial torsion, which is common in achondroplasia, is difficult to address with guided growth, and guided growth cannot be used in patients with closed physes. In these situations, osteotomy will be necessary.

Lower extremity procedures were performed at a younger age in four different geographic locations. Similarly, lower extremity osteotomies, the most common lower extremity surgery, was also performed at a younger age in all four centers. Interestingly, a smaller second peak in lower extremity procedures was detected between 40 and 60 years of age at three centers. One of the four centers included in this study was a pediatric hospital; therefore, a second peak in lower extremity procedures was not expected at this particular center. Lower extremity osteotomies were predominantly performed at a younger age and did not contribute to the second peak in the bimodal distribution of lower extremity procedures. This analysis did not try to determine which type or types of fixation were used following osteotomies.

The second peak in lower extremity procedures between 40 and 60 years of age was related to surgery performed in nine patients. The Kaplan Meier analysis revealed this second peak due to the relatively small number of patients in this age group: the cohort born prior to 1980 had 17% (234/1374) of the entire sample. Although three of these patients in the 40-to-60-year age group received total hip arthroplasty and one patient received total knee arthroplasty, the overall frequency of hip and knee arthroplasty in the achondroplasia cohort was much smaller than the general population. In an analysis of the Healthcare Cost and Utilization Project State Inpatient Databases and the National Hospital Discharge Survey of the general population, Kremers et al. found a prevalence of 0.83% for total hip arthroplasty and 1.52% for total knee arthroplasty in the United States [39]. Once again, this finding may relate to the relatively young ages of patients within our cohort. This finding may also reflect the smaller mechanical forces at the hip and knee in patients with achondroplasia. In addition, mouse studies demonstrate a protective mechanism against osteoarthritis for the *FGFR3* mutation [40, 41].

The consistency of the timing of lower extremity procedures, including lower extremity osteotomies, across centers in different parts of the country stands in contrast to previous study of orthopedic procedures demonstrating geographic variation. To examine geographic trends in revision and primary hip and knee arthroplasty, Hilibrand et al. utilized the Nationwide Inpatient Sample and found a 2.2-fold variation and 2.1-fold variation in the revision rate ratio by state for revision total knee arthroplasty and revision total hip arthroplasty [42]. The authors concluded that significant variation exists in the performance of revision total knee and hip arthroplasty procedures from state to state. In another study examining primary total hip and knee arthroplasty performed in England, Judge et al. found that significant geographic variation existed across districts, controlling for distance measures [43]. Demographic variables contributed to variation in some districts while other districts were not influence by these factors. They concluded that the geographic variation in joint replacement surgery needs to be further delineated to facilitate access for all patients.

The consistency in the timing of lower extremity surgery in patients with achondroplasia may be due to the small number of specialists performing these procedures. In this relatively small dysplasia community, consensus likely exists in performing lower extremity surgery, particularly lower extremity osteotomies, in younger patients. In contrast, arthroplasty surgery is performed by a large number of surgeons who may have diverging views on indications and timing of surgery.

The highest incidence of lower extremity procedures, including lower extremity osteotomies, was performed in the cohort born between 1990 and 1999. Interestingly, the oldest patients born prior to 1980 had the lowest incidence of lower extremity procedures, suggesting that lower extremity interventions were performed more frequently in the last three decades with an increase from 14.21 surgeries per 1,000 person years in the cohort born prior to 1980 to 69.96 surgeries per 1,000 person years in the cohort born between 1990 and 1999.

The increase in the number of lower extremity procedures performed more recently in patients with achondroplasia is consistent with a general trend in orthopedics for overall increase in number of procedures. In a study of arthroscopic knee surgery performed in England between 1997 and 2017, Abram et al. found that the incidence of arthroscopic partial meniscectomy increased from 51/100,000 in 1997–1998 to 120/100,000 in 2016–2017 [44]. In addition, they found that the incidence of arthroscopic chondroplasty increased from 3.2/100,000 in 1997–1998 to 51/100,000 in 2016–2017. The trend toward more frequent procedures in orthopedics is predicted to continue to increase. In a study of the number of hip arthroscopies performed by the National Health Service in England, Palmer et al. predicted a 1388% increase in the number of hip arthroscopies performed in 2023 as compared to 2002 [45].

Risk factors for repeat spine surgery included increased weight and height. The theme of increased patient size leading to worse outcomes and/or repeat surgical intervention is prevalent in orthopedic surgery [46-49]. In a study of patients undergoing orthopedic trauma procedures, the complication rate in obese patients was 38% compared to 28% (p = 0.03) in non-obese patients [50]. The authors concluded that obese orthopedic trauma patients are at higher risk for in hospital complications with further study required to optimize results. In another analysis of failed total hip arthroplasty, Goodnough et al. found that the rate of aseptic loosening leading to failure of primary total hip arthroplasty was 30% in obese patients and 18% in non-obese patients [51]. The authors found a similar increase in infected failed total hip arthroplasty among obese patients.

This analysis demonstrates consistency in the timing of lower extremity procedures across all four centers and a trend towards more frequent lower extremity procedures in recent decades. Finally, malalignment is a consistent indication for surgery for first time as well as repeat lower extremity surgery. Future study focusing on decreasing the number of repeat lower extremity surgery should be performed. Nevertheless, several meaningful observations are drawn in this study for orthopedic surgeons managing the complex lower extremity deformities observed in patients with achondroplasia.

### Limitations

The major limitation of this study is its clinic-based and retrospective nature. The number of individuals is skewed very heavily towards the younger decades. The data which could be extracted into the database is limited by what was available in the charts reviewed. Specific details such as the precise indications and outcomes were limited by what was documented and there was no specific predefined criteria. The main strengths of our study findings are the size of this natural history cohort, the follow-up of over four decades, and the uniform data collection by medical providers with extensive achondroplasia care experience.

## Conclusions

Orthopedic surgery was a common occurrence in achondroplasia with 29.7% of patients undergoing at least one orthopedic procedure. Spine surgery (12.7%) was less common and occurred at a later age than lower extremity surgery (21.2%). In subjects reaching 40 years of age, overall 60.9% had an orthopedic procedure with 35.3% having a lower extremity surgery and 39.6% with spine surgery. Cervicomedullary decompression and hydrocephalus with shunt placement were associated with an increased risk for spine surgery. The results from CLARITY, the largest natural history study of achondroplasia, represents the practices of 4 specialized US skeletal dysplasia centers over 6 decades and may be used by clinicians in counseling patients and families about orthopedic surgery.

## Methods

CLARITY was reviewed and approved by the Institutional Review Board at each of the participating centers. Four centers participated in the study and contributed patients to the PAC, which represents the retrospective arm of CLARITY. Study sites were Johns Hopkins University, Baltimore, MD; Nemours/A.I. duPont Hospital for Children, Wilmington, DE; McGovern Medical School UTHealth, Houston, TX; and University of Wisconsin School of Medicine and Public Health, Madison, WI.

Methodology regarding collection of data at each institution was previously described [17]. In brief, variables of interest collected for CLARITY were related to mixed longitudinal anthropometry, polysomnography and sleep disordered breathing, radiographic catalogue, and surgical burden. Data were collected and stored in the Research Electronic Data Capture (REDCap) system managed at JHU.

Within the surgical domain, five achondroplasia-related surgical categories were included: otolaryngology (including tonsillectomy/adenoidectomy and ear tubes), brain (including shunt and ventriculostomy), cervicomedullary decompression (CMD, which included decompression of the foramen magnum with or without laminectomy of C1 or C1 and C2), spine, and lower extremity procedures. This study focused on orthopedic intervention, including spine (cervical laminectomy below C2, thoracic and lumbosacral laminectomy and spinal fusion) and lower extremity procedures (osteotomy, tension band plating for guided growth, fibulectomy, limb lengthening, epiphysiodesis, hip replacement, implant removal).

Descriptive statistics were reported as frequencies (proportions) for categorical variables. Mean values (standard deviations (SD)) were utilized for normally distributed continuous variables and median values (ranges) were utilized for non-normally distributed continuous variables. Kaplan Meier curves were generated to describe the time-to-event of spine and lower extremity surgeries. Curves and calculate the probability to be free from surgery at each time interval using the *survival* package in R version 4.1.2. Kaplan Meier plots were generated using the package *survminer*. The ‘failure’ time or the time-to-event was calculated by the age at first spine and lower extremity surgery, and the ‘censor’ time was calculated by the age at the last known medical contact for people who did not have the surgery. Odds ratios (OR) (with 95% confidence intervals (95% CI)) were calculated to illustrate odds of spine and lower extremity surgery occurring after CMD and shunting as well as other risk factors in this achondroplasia cohort. Odds ratios were calculated by logistic regression. *p* < 0.05 was considered statistically significant. Statistical analysis was performed with R version 4.1.2.

We examined the association of anthropometric measures and the need for an orthopedic procedure. The closest measurements to the procedure date of height in centimeters, weight in kilograms and OFC in centimeters when within one month of the procedure were explored using logistic regression. These ORs illustrate the relative odds of surgery per one unit increase in growth.

## Supplementary Information

**Additional file 1: Figure S1**. Kaplan Meier curve for spine procedures performed by center. Spine surgeries happened later in life in three of four centers that treat adult patients. No patients older than 35 years of age received surgery at duPont.

**Additional file 2: Figure S2**. Kaplan Meier curve for lower extremity procedures performed by center. Most lower extremity procedures were performed before the age of 20 years old at each center. A second cluster of lower extremity procedures was performed in older patients at two centers.

## Data Availability

The datasets generated and/or analyzed during the current study are not publicly available but are available from the corresponding author on reasonable request.

## Abbreviations

CI: Confidence Interval
CLARITY: The Achondroplasia Natural History Study
CMD: Cervicomedullary decompression
CPAP: Continuous Positive Airway Pressure
FGFR3: Fibroblast Growth Factor Receptor-3
OFC: Occipital Frontal Circumference
OR: Odds Ratio
OSA: Obstructive Sleep Apnea
PAC: Primary Achondroplasia Cohort
REDCap: Research Electronic Data Capture
SD: Standard Deviation
TLK: Thoracolumbar Kyphosis
