# Supplementary figures and images for: Achondroplasia natural history study (CLARITY): 60-year experience in orthopedic surgery from four skeletal dysplasia centers
Source: Orphanet J Rare Dis. 2023 Jun 6;18:139. doi: 10.1186/s13023-023-02738-x (PMC10246371; doi:10.1186/s13023-023-02738-x)

## Slide 1
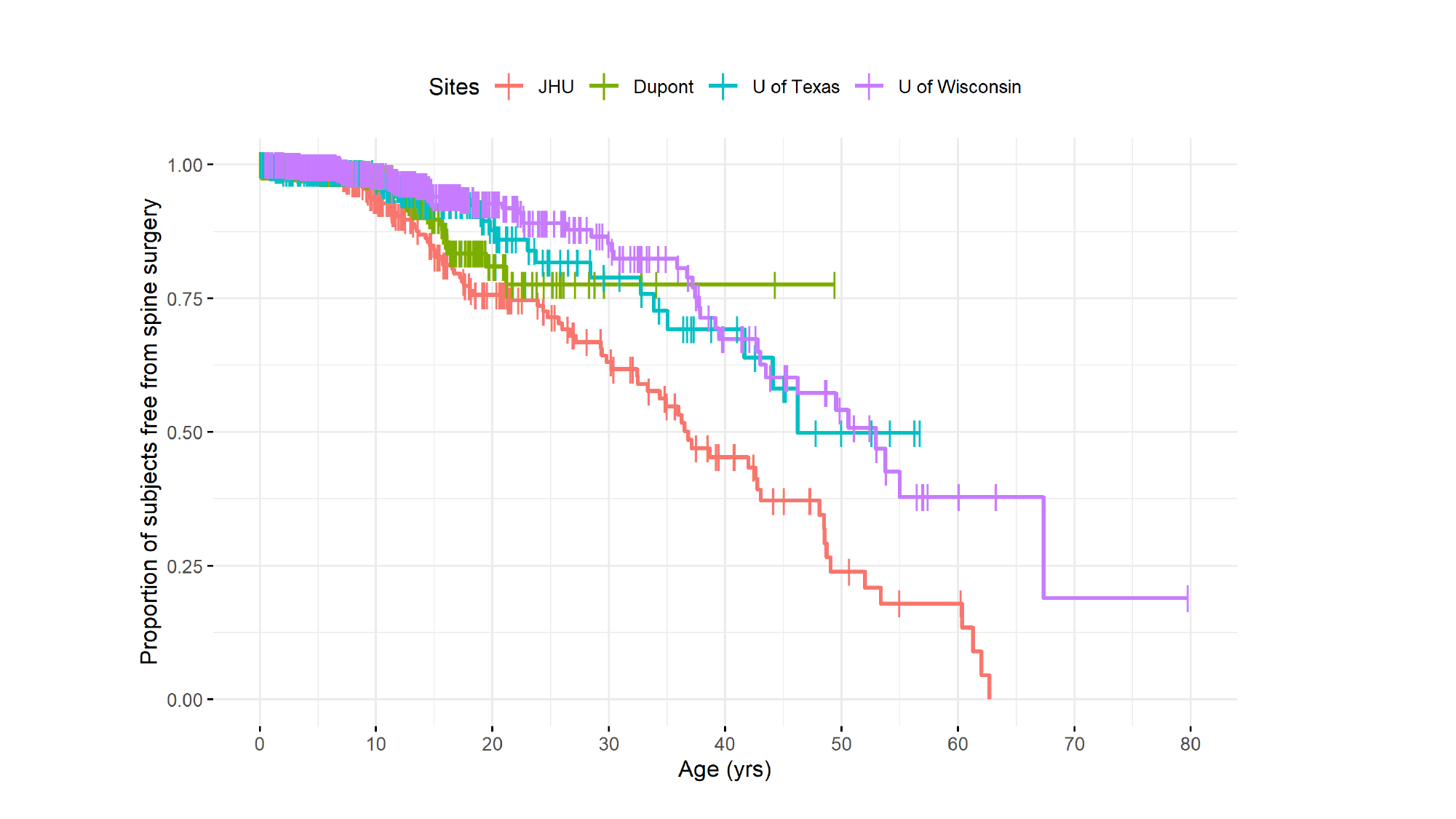

Supplement: Supplementary file 1 — Additional file 1: Figure S1. Kaplan Meier curve for spine procedures performed by center. Spine surgeries happened later in life in three of four centers that treat adult patients. No patients older than 35 years of age received surgery at duPont. [file 13023_2023_2738_MOESM1_ESM.pptx]

## Slide 1
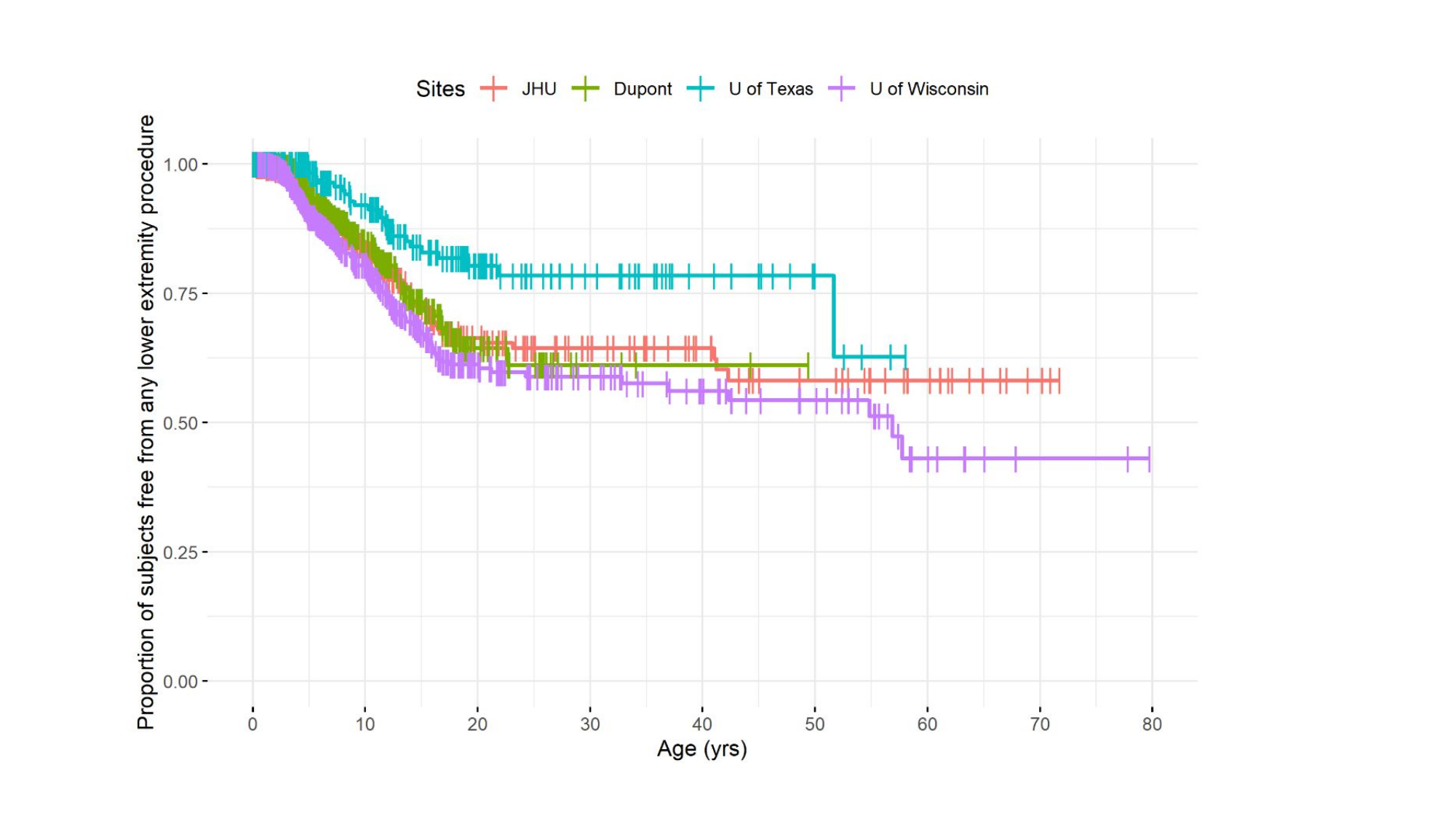

Supplement: Supplementary file 2 — Additional file 2: Figure S2. Kaplan Meier curve for lower extremity procedures performed by center. Most lower extremity procedures were performed before the age of 20 years old at each center. A second cluster of lower extremity procedures was performed in older patients at two centers. [file 13023_2023_2738_MOESM2_ESM.pptx]
